# Comparison of Piezosurgery and Conventional Rotary Instruments for Removal of Impacted Mandibular Third Molars: A Randomized Controlled Clinical and Radiographic Trial

**DOI:** 10.1155/2016/8169356

**Published:** 2016-08-14

**Authors:** Hani Arakji, Mohamed Shokry, Nayer Aboelsaad

**Affiliations:** ^1^Oral Surgical Sciences Department, Division of Oral and Maxillofacial Surgery, Faculty of Dentistry, Beirut Arab University, Riad El Solh, P.O. Box 11-5020, Beirut 11072809, Lebanon; ^2^Oral Surgical Sciences Department, Faculty of Dentistry, Beirut Arab University, Riad El Solh, P.O. Box 11-5020, Beirut 11072809, Lebanon; ^3^Oral and Maxillofacial Surgery Department, Faculty of Dentistry, Alexandria University, Alexandria, Egypt; ^4^Oral Medicine and Periodontology Department, Faculty of Dentistry, Mansoura University, Mansoura 35516, Egypt

## Abstract

The purpose of this study was to test the effect of the surgical removal of impacted mandibular third molars using piezosurgery versus the conventional surgical technique on postoperative sequelae and bone healing.* Material and Methods. *This study was carried out as a randomized controlled clinical trial: split mouth design. Twenty patients with bilateral mandibular third molar mesioangular impaction class II position B indicated for surgical extraction were treated randomly using either the piezosurgery or the conventional bur technique on each site. Duration of the procedure, postoperative edema, trismus, pain, healing, and bone density and quantity were evaluated up to 6 months postoperatively.* Results. *Test and control sites were compared using paired* t*-test. There was statistical significance in reduction of pain and swelling in test sites, where the time of the procedure was statistically increased in test site. For bone quantity and quality, statistical difference was found where test site showed better results.* Conclusion. *Piezosurgery technique improves quality of patient's life in form of decrease of postoperative pain, trismus, and swelling. Furthermore, it enhances bone quality within the extraction socket and bone quantity along the distal aspect of the mandibular second molar.

## 1. Introduction

Approximately 20% of the population has impacted teeth, where mandibular and maxillary third molars are the most common [1].

The highest incidence of impaction has been shown in mandibular wisdom teeth which may lead to pathologies like pericoronitis, periodontitis, second molars tooth-crown resorption, pain, cysts or odontogenic tumors, and primary or secondary crowding of the dentition. Early removal of these teeth to prevent the abovementioned problems is widely approved [2].

Surgical removal of these teeth is usually correlated with postoperative pain, swelling, and trismus whereas complications such as infection, dry socket, trigeminal nerve injuries, and, rarely, fracture of the mandible are less common to occur [1, 3].

Hard tissue cutting is a common procedure in the dental fields, especially during maxillofacial, oral, and periodontal surgeries. Traditionally, rotating instruments like burs have been used for osseous surgery. However, bone overheating and damage to adjacent tissues are disadvantages that are related to the use of these methods [4].

Piezosurgery is a novel technique that has been introduced as a valuable alternative to overcome the disadvantages associated with the conventional rotating bone cutting instruments [4]. It is performed by means of a device that uses microvibration at a frequency capable of cutting bone. Its mechanism of action is based on the ability of certain ceramics and crystals to deform when an electric current is passed across them, resulting in microvibration at ultrasonic frequency [5]. A frequency of 25–30 KHz, from a nitride-hardened or diamond-coated insert, allows for selective cut of bone tissue [6].

Since its approval for commercial use in 2002, it has been successfully utilized for many surgical procedures, such as maxillary sinus lifting, autologous bone graft harvesting, bone splitting, lateralization of the inferior alveolar nerve, and orthognathic and neurologic surgeries [7–9].

Goyal et al. (2012) [10] compared piezosurgery with the conventional rotary surgical technique and found that pain, swelling, trismus, and healing were significantly decreased in the piezosurgery site. Moreover, a systematic review and meta-analysis done by Jiang et al. 2015 [11] compared piezosurgery and conventional rotary osteotomy techniques in third molar extraction. They concluded that although the patients undergoing piezosurgery experienced longer surgery time, they developed less swelling, less pain, and less postoperative trismus.

Also, similar findings were concluded in the meta-analysis conducted by Al-Moraissi et al. 2015 [12] in which there was a significant reduction in postoperative sequelae (facial swelling, pain, and trismus) with the piezoelectric surgical technique in third molar extraction, whereas their results showed that the duration of surgery and operating time for third molar extraction were significantly shorter with conventional rotary instruments compared to the piezoelectric surgical technique.

Despite all the studies that had been conducted similar to this present work, they did not tackle the piezosurgery as a recent bone cutting method on bone quality and bone quantity on the resultant extraction site; therefore this study was performed in order to evaluate the bone healing within the extraction socket after removal of the impacted mandibular third molar using piezosurgery together with its effect on the postoperative sequelae: pain, trismus, and swelling.

## 2. Materials and Methods

### 2.1. Study Design and Setting

This study was carried out as a randomized controlled clinical trial: split mouth design. The estimated sample size was calculated according to http://epitools.ausvet.com.au/, by taking the means of mouth opening from a previous similar study conducted by Goyal et al. [10] where mean for test site = 2.54 ± (0.93) and mean for control site = 3.91 ± (0.99), where the variance was calculated to be 0.92, assuming a confidence level of 95% and a study power of 80%. The calculated sample size was 16 male patients (32 operating sites). 20% was added to the sample size from the start of the study to eliminate the probability of drop-out through the treatment protocol. Therefore, twenty male patients (40 operating sites) who required removal of bilateral impacted lower third molars were conveniently recruited from the outpatient Department of Oral Surgical Sciences, Faculty of Dentistry, Beirut Arab University, Beirut, Lebanon. They have been selected to fulfill certain inclusion and exclusion criteria. The inclusion criteria were male patients with age ranging from 18 to 35 years having bilateral mandibular mesioangular impacted third molars (Pell and Gregory class II, position B) [13]. On the other hand, the exclusion criteria were heavy smokers (≥25 cigarettes) [14], uncontrolled systemic conditions, pathologies, and infection related to the site of surgery.

Sites were randomly selected by tossing a coin, where the face was the test site where piezosurgery (Mectron Dental, Italy) was performed and the back was the control site where the conventional rotary instruments have been used.

All work was conducted in accordance with the Declaration of Helsinki (1964), where all patients were informed about the whole procedure and signed a detailed consent form. The study had been started after the approval of the Institutional Review Board (IRB) of Beirut Arab University, code 2015H-025-D-M-0085.

### 2.2. Preoperative Phase

A detailed medical and dental history and panoramic and intraoral periapical radiographs of the surgical site were taken. The patients were instructed to rinse their mouth by chlorhexidine mouthwash 0.12%, 30 minutes before the operation.

### 2.3. Surgical Phase

**Figure 1:**
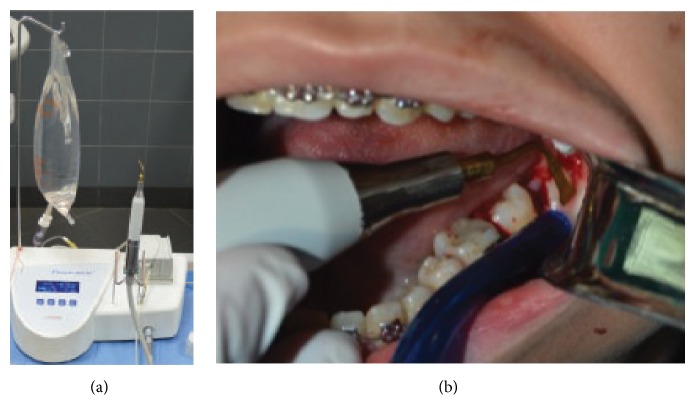
(a) Piezosurgery device and (b) bone guttering around impacted mandibular third molar using piezosurgery (test site).

All operations were done by the same surgeon under local anesthesia consisting of 2% lidocaine hydrochloride with 1 : 80,000 adrenaline (Lignospan Special, Septodont, UK). Both sites were prepared with 5% povidone-iodine solution, and a conventional extended buccal incision was made. A mucoperiosteal flap was reflected with a periosteal (Molt number 9) elevator to expose the impacted tooth and surrounding bone. For control site, a number 6 carbide round bur (DENTSPLY, USA) mounted on a straight handpiece was used at 35,000 rpm for guttering at the buccal and distal aspect of the tooth. A straight fissure bur was used to section the tooth. At all times cutting of bone and tooth was accompanied by copious irrigation with cooled saline solution. For test site, the OT7 and OT2 (Mectron Inserts, Italy) cutting inserts of the piezosurgery were used for bone guttering around the impacted tooth (Figure 1). The frequency was adjusted between 28 and 36 kHz and the microvibration amplitude between 30 and 60 *μ*m/s. Sectioning of the teeth has been performed in the same manner as the control site. In both sites after removal of the tooth the extraction socket was debrided, irrigated with 0.9% normal saline, and closed with 3/0 black silk sutures.

### 2.4. Postsurgical Phase

The duration of the operation was calculated in each case from the start of the incision till the termination of the suturing. Patients were instructed to apply cold fomentation for 10 min/hour for the first 6 postsurgical hours. Amoxicillin with clavulanic acid 625 mg (Augmentin by GlaxoSmithKline), 3 times daily for 5 days, and ibuprofen 400 mg (Bruffen Abbott) twice daily for 3 days were prescribed. Sutures were removed after 7 days.

### 2.5. Follow-Up Phase

#### 2.5.1. Clinical Variables

Pain, trismus, and swelling were evaluated on days 1, 7, and 14 postoperatively. Also healing of the flap and color of the overlying mucosa were checked.

Postoperative pain was assessed using a visual analogue scale (VAS) [2]. Trismus was evaluated by measuring the interincisal distance between the incisal edge of the upper and lower central incisors using a caliper at maximum mouth opening (cm). Furthermore, postoperative swelling was measured using a tape by taking the mean of the distance between the lateral corner of the eye and the angle of the mandible, tragus and the outer corner of the mouth, and tragus and soft tissue pogonion [2].

#### 2.5.2. Radiographical Variables

**Figure 2:**
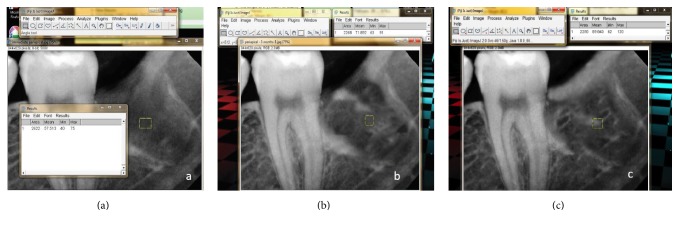
(a) Immediate, (b) 3-month, and (c) 6-month standardized periapical X-ray measuring bone density using ImageJ software (test site).

Standardized periapical X-rays using the XCP (RINN, DENTSPLY, USA) sensor holder were taken on the same day of surgery as a baseline, 3 months and 6 months postoperatively, in order to measure bone density using ImageJ software (image processing and analysis in Java V. 1.48). A standardized sized square (33×33 pixels) was inserted in the center of the extracted socket which is determined by identification of the intersected point between 2 straight lines: a horizontal line extending from the anterior border of the ramus to a midpoint on the distal aspect of the mandibular second molar and a vertical line extending from the alveolar bone crest to the roof of the inferior alveolar canal. The bone density within this square was measured by selecting Region of Interest (ROI), from tools, and then the given data was analyzed in terms of pixels (Figures 2(a)–2(c)). The same square was drawn for each X-ray for all patients where the bone density was measured [15].

**Figure 3:**
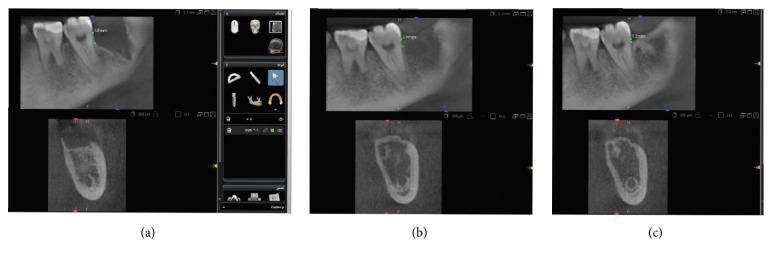
(a) Immediate, (b) 3-month, and (c) 6-month CBCT showing bone height measurement along the distal aspect of the second molar (test site).

CBCT (CS 9300, Carestream, USA) were taken immediately, 3 and 6 months postoperatively (Figures 3(a)–3(c)), for measurement of marginal bone height along the distal aspect of the mandibular second molar through drawing a straight line extending from the cementoenamel junction (CEJ) of the distal aspect of the mandibular second molar to the alveolar bone crest [16].

Test and control sites were compared regarding the study clinical and radiographic variables using paired *t*-test. Significance level was set at the 5% level. Statistical analysis was performed using SPSS version 20.0.

## 3. Results 

Twenty male patients who had bilateral impacted mandibular third molars extracted were included in the study. Their age ranged between 19 and 32 years. All 20 patients completed the 6-month follow-up period with no drop-out from the sample.

The mean ± (SD) time of surgery was 17.60 ± (2.95) min in control site, whereas it was 28.50 ± (3.57) min in the test site. When comparing both sites regarding the operation time there was a statistical difference (*P* = 0.0001).

All patients were thoroughly clinically evaluated starting from the first postoperative day till the 14th postoperative day. They showed an eventful soft tissue healing with absence of any signs of infection.

Table 1 shows the comparison of pain sensation measured by VAS score between test and control at different follow-up periods. Significant differences existed in mean VAS scores after 1, 7, and 14 days (*P* ≤ 0.0001, <0.0001, and 0.001). After 1, 7, and 14 days, mean VAS scores in the test site were lower than that in the control site (mean in test = 3.60, 1.10, and 0.10 compared to mean in control = 6.70, 3.30, and 1.00).

Moreover, Table 2 shows the comparison of trismus between test and control at different follow-up periods. Significant differences existed between mean measurements, indicating mouth opening at baseline and after 1, 7, and 14 days (*P* ≤ 0.0001, <0.0001, <0.0001, and 0.002). At baseline and after 1, 7, and 14 days, mouth opening in the control sites was less than at test sites (mean in control = 4.50, 2.74, 3.49, and 4.49 compared to mean in test = 4.78, 3.85, 4.53, and 4.77).

Also, Table 3 shows the comparison of swelling between test and control at different follow-up periods. Significant differences existed between mean measurements indicating swelling at 1, 7, and 14 days postoperatively (*P* ≤ 0.0001, <0.0001, and 0.03). At 1, 7, and 14 days postoperatively, swelling was greater at control sites than at test sites (mean in control = 12.32, 11.78, and 11.30 compared to mean in test = 11.55, 11.29, and 11.20).

**Figure 4:**
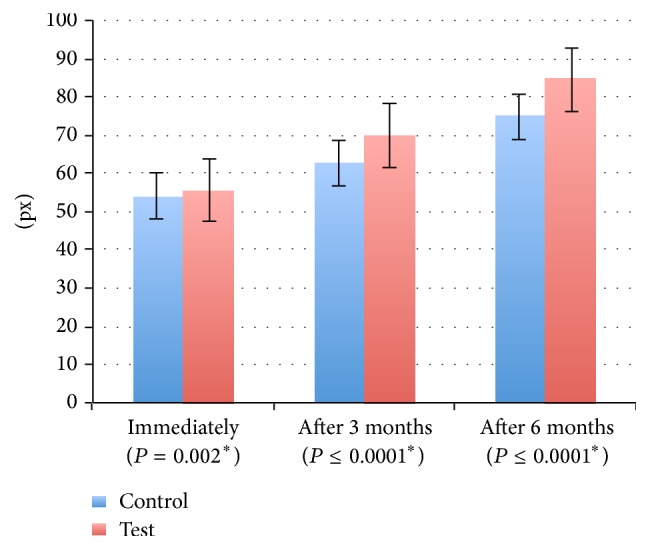
Mean and standard deviation for bone density (pixels). *∗*: statistically significant at *P* ≤ 0.05.

Radiographically, the mean bone density in test site immediately and after 3 months and 6 months postoperatively, respectively, was 55.70 ± (3.60), 69.80 ± (8.19), and 84.45 ± (4.73), where the control recorded 54.00 ± (3.87), 62.75 ± (5.19), and 74.87 ± (4.03) in the same interval period. The results showed statistical difference between the two sites where piezosurgery site showed improved bone quality (*P* ≤ 0.0001) (Figure 4).

**Figure 5:**
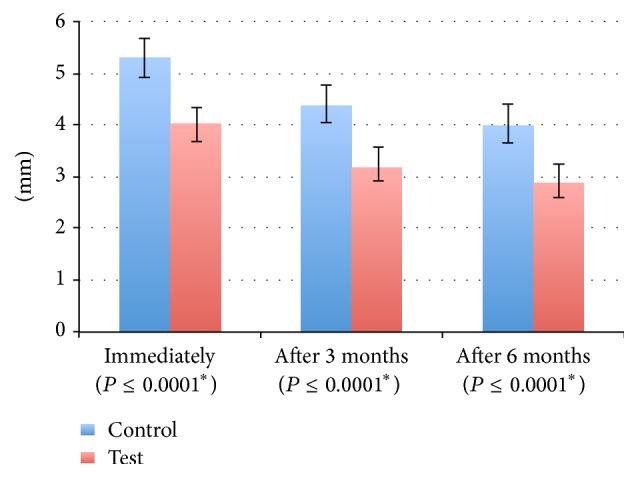
Mean and standard deviation for bone loss (millimeters). *∗*: statistically significant at *P* ≤ 0.05.

On the other hand, bone loss has been observed along the distal aspect of the second molar within the two sites; greater amount of bone loss was statistically noticed in the control site when it was compared to the test site (*P* ≤ 0.0001). Immediately after the operation and after 3 and 6 months, mean bone loss in the control site was greater than that in the test site (mean in control = 5.30, 4.41, and 4.03 compared to mean in test = 4.01, 3.23, and 2.91) (Figure 5).

## 4. Discussion

Recently, after painstaking research and the application of advanced principles of physics, newer instruments have been introduced to reduce the difficulty and morbidity in third molar surgery. One such innovation is piezosurgery or the application of piezoelectric, ultrasonic vibrations to make precise and safe osteotomies [17].

This study was carried out as an experimental, randomized, controlled clinical trial: split mouth design; this type of study is especially selected as it has the distinct advantage of removing the patients compliance bias from the estimated treatment effect as described by Zhu et al. [18].

To standardize our results, it was conducted on twenty male patients having their age ranging from 19 to 32 years, in order to remove the gender factor that may play a role in postoperative complications due to hormonal changes in females. They had mesioangular class II position B bony impacted mandibular third molar, according to Pell and Gregory [13]. This type of impaction was selected as it is the most commonly found and it was in agreement with a study conducted by Goyal et al. [10] and Piersanti et al. [17] where they chose the same impacted mandibular third molar category in their study.

There was no drop-out from the selected sample and this may be attributed to the well-educated level of the selected patients and their commitment to their treatment in addition to the availability of the social media which makes the follow-up communication with the patients easier.

The duration of the procedure in each site was calculated in terms of minutes starting from the establishment of the flap till the end of suturing. The mean duration of the operation was longer in the piezosurgery site than in the control site. This is in agreement with a similar study performed by Goyal et al. [10].

The mean recorded pain score was significantly lesser in the study site than in the control site. This finding is parallel to the results obtained by Goyal et al. [10], Mantovani et al. [19], and Piersanti et al. [17]. They reported in their studies a significant difference in pain score using the same scale, and all agreed that the site where the impacted mandibular third molar resides using piezosurgery has less postoperative pain.

Furthermore swelling was evaluated in this study. Better improvement was noticed within the test site and this is in accordance with studies done by Pappalardo and Guarnieri [20], Mantovani et al. [19], Piersanti et al. [17], and Mozatti et al. [21] where they compared the postoperative outcomes between piezosurgery and conventional rotary surgery in removing mandibular third molars.

These results run along the same line of findings of a meta-analysis study conducted by Jiang et al. [11] where 7 studies were included in their analysis. The aim of their study was to compare piezosurgery with rotary osteotomy techniques, with regard to surgery time and the severity of postoperative sequelae, including pain, swelling, and trismus. Their meta-analysis indicates that although patients undergoing piezosurgery experienced longer surgery time, they had less postoperative swelling, indicating that piezosurgery is a promising alternative technique for extraction of impacted third molars.

Radiographically, bone density was assessed by the aid of standardized periapical radiographs. While the literature supports the usefulness of CBCT scans for the determination of radiographic bone density [22–25], there are other studies stating that the grey levels in CBCT scans are not accurate when compared with CT. In a 2006 presentation, Armstrong [26] concluded that “Hounsfield units sampled from identical anatomic areas with CBCT and Medical CT (MDCT) are not identical.” A study carried out by Katsumata et al. [27] found that the grey levels in a CBCT image for bone varied from 1500 to over 3000. They concluded that “the ability to assess the density or quality of bone is limited and because the grey level range is so variable the derived density provided less than meaningful data.”

Due to all the previous factors, we selected the standardized digital radiography for assessing the bone density in an accurate manner.

Standardization of the periapical X-rays was done through silicone based bite block for each patient for purpose of repeatability of the position of the sensor; also the angulation of the cone was standardized using the RINN XCP paralleling device.

It was showed that there is a greater increase in bone density occurring within the test site from immediately postoperative period to 6 months after surgery. These results are in accordance with Vercelotti et al. [28] where they compared piezosurgery with carbide burs in ostectomy and osteoplasty and proved that there is better bone healing in terms of quantity and quality when using piezosurgery in osseous surgeries. Moreover, Rullo et al. [29] analyzed the bone histology and found well-defined histological differences between the bone samples collected with the bur and the ultrasonic device. They reported that more integrity of the bony structure, well-designed osteotomy lines, and no evidence of bone heat osteonecrosis characterized the bone samples harvested with the piezoelectric device.

The alveolar bone loss was assessed radiographically using CBCT. There was a greater loss in bone height along the distal aspect of the mandibular second molar in the control site than in test site from baseline to 6 months after surgery. This difference was statistically significant. Rahnama et al. [30] stated that the ultrasound vibration stimulates cells' metabolism. Moreover, the lack of necrosis in the cut area accelerates bone regeneration. Soft tissue damage is not noticed. Furthermore, Labanca et al. [31] have made a review on piezosurgery and found that it has less damage to osteocytes and this can explain the decreased bone loss within the test site compared to the control one. Taking into consideration the aforementioned observations and despite the presence of controversies about the effect of surgical removal of impacted mandibular third molar on periodontal health distal to the adjacent second molar [32], the current study has shown a decreased bone loss along the distal aspect of the mandibular second molar when using piezosurgery, which enhances the periodontal health condition along the distal aspect of the second molar reducing the need for performing further periodontal procedures. Similarly, Tsai et al. [33] stated that piezoelectric surgical instruments might promote faster wound healing compared to rotary instruments over a short-term observation period.

The main disadvantage of piezosurgery noticed so far besides expense and the risk of breakage of the surgical tips is the increased operating time as a result of the slow rate of cutting. The time of surgery can be improved by the operator's experience. Increasing the sample size with longer duration of follow-up and taking bone specimen for histological examination from the surgical site can add valuable findings to the previous results.

## 5. Conclusion

Within the limitation of this study, it can be concluded that piezosurgery reduces postoperative pain, trismus, and swelling and enhances the postsurgical quality of patient's life. Also, it may play an important role in increasing bone density within the extraction socket and decreasing the amount of bone loss along the distal aspect of the mandibular second molar.

## Figures and Tables

**Table 1:** Comparison of pain sensations between study and control at different follow-up periods.

	Mean (SD)		*P* value of paired *t*-test
	Study	Control	
After 1 day	3.60 (1.71)	6.70 (0.95)	<0.0001^*∗*^
After 7 days	1.10 (0.74)	3.30 (0.95)	<0.0001^*∗*^
After 14 days	0.10 (0.32)	1.00 (0.67)	0.001^*∗*^
*P* value of paired *t*-test	<0.0001^*∗*^	<0.0001^*∗*^	

^*∗*^Statistically significant at *P* ≤ 0.05.

**Table 2:** Comparison of trismus (mouth opening) between test and control at different follow-up periods (in millimeters).

	Mean (SD)		*P* value of paired *t*-test
	Test	Control	
Baseline	4.78 (0.14)	4.50 (0.11)	<0.0001^*∗*^
After 1 day	3.85 (0.07)	2.74 (0.13)	<0.0001^*∗*^
After 7 days	4.53 (0.08)	3.49 (0.09)	<0.0001^*∗*^
After 14 days	4.77 (0.22)	4.49 (0.11)	0.002^*∗*^
*P* value of paired *t*-test	0.61	0.81	

^*∗*^Statistically significant at *P* ≤ 0.05.

**Table 3:** Comparison of swellings between test and control at different follow-up periods (in millimeters).

	Mean (SD)		*P* value of paired *t*-test
	Test	Control	
Baseline	11.21 (0.07)	11.27 (0.05)	0.001^*∗*^
After 1 day	11.55 (0.08)	12.32 (0.04)	<0.0001^*∗*^
After 7 days	11.29 (0.08)	11.78 (0.12)	<0.0001^*∗*^
After 14 days	11.20 (0.04)	11.30 (0.15)	0.03^*∗*^
*P* value of paired *t*-test	0.43	0.46	

^*∗*^Statistically significant at *P* ≤ 0.05.
